# Ginsenoside Rg3 inhibits the biological activity of SGC‐7901

**DOI:** 10.1002/fsn3.1707

**Published:** 2020-06-24

**Authors:** Qing Yang, Ning Cai, Daobiao Che, Xing Chen, Dongliang Wang

**Affiliations:** ^1^ Department of Hospital Pharmacy Suqian First Hospital Suqian China; ^2^ Emergency Department Suqian First Hospital Suqian China

**Keywords:** AKT, biological activities, gastric cancer, ginsenoside Rg3, p‐PI3K, PTEN, SGC‐7901

## Abstract

**Aim:**

To explore the suppressive effects of ginsenoside Rg3 on the biological activities of gastric cancer and the mechanisms responsible therein, by conducting an in vitro study.

**Materials and Methods:**

SGC‐7901 gastric cancer cells were divided into NC, DMSO, Gin‐Low (10 mg/L), Gin‐Middle (20 mg/L), and Gin‐High (40 mg/L) groups. Using MTT, flow cytometry, transwell, and wound‐healing assays, the cell biological activities in the different groups were evaluated; the protein expression levels of PTEN, p‐PI3K, AKT, and P53 were measured by Western blot assay, and p‐PI3K nuclear volume was evaluated by immunofluorescence.

**Results:**

The SGC‐7901 cell proliferation rate was depressed significantly, and cell apoptosis increased significantly while cells were arrested in the G1 phase (*p* < .05) with ginsenoside Rg3 treatment in a dose‐dependent manner (*p* < .05). Meanwhile, the SGC‐7901 cell invasion number and wound‐healing rate of ginsenoside Rg3‐treated groups were significantly downregulated compared with those of the NC group, also in a dose‐dependent manner (*p* < .05). PTEN and P53 protein expression levels were significantly increased, and p‐PI3K and AKT protein expression levels were significantly depressed in ginsenoside Rg3‐treated groups in a dose‐dependent manner (*p* < .05).

**Conclusion:**

Ginsenoside Rg3 suppresses gastric cancer via regulation of the PTEN/p‐PI3K/AKT pathway.

## Graphical abstract

Ginsenoside Rg3 suppressed gastric cancer via regulation PTEN/p‐PI3K/AKT pathway.

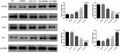

## 1 INTRODUCTION

Gastric cancer is a highly prevalent malignancy of the digestive system with high morbidity and mortality (Siegel, Miller, & Jemal, 2018). The incidence of gastric cancer in China is high, as demonstrated by data from National Cancer Center of China; gastric cancer mortality was 498/100,000 in China, over twice the global average, ranking second among all malignancies in China (Chen et al., 2016). Currently, gastric cancer is mainly treated with surgery, radiotherapy, chemotherapy, interventional therapy, and biological therapy. Commonly used antitumor drugs such as 5‐fluorouracil, cytarabine, and doxorubicin can alleviate symptoms, control tumor biological activities, and prolong survival, but they are very expensive, highly toxic and prone to drug resistance; moreover, they can impair immunity, bone marrow function, and digestive function. Therefore, antitumor drugs with high efficiency and low toxicity are highly desirable. Among the traditional Chinese medicines, ginseng is a valuable herb that shows a wide range of pharmacological effects (Shan et al., 2015; Zhang et al., 2012). Studies have shown that ginsenoside Rg3 can suppress various cancer cell biological activities (Chang, Huo, Lv, Wang, & Liu, 2014; Cheong et al., 2015; Cho, Kim, Kim, Ha, & Ahn, 2014; Joo et al., 2015; Yoon et al., 2015; Yu et al., 2015), but the relevant mechanisms have not been clarified, and reports on the effects of ginsenoside Rg3 on gastric cancer cell biological activities are rare. In the present research, we used SGC‐7901 as the model system and investigated the effects of ginsenoside Rg3 on the biological activities of intestinal cancer cells and the possible mechanism of its action by MTT assay, transwell assay, wound‐healing assay, and Western blotting.

## 2 MATERIALS AND METHODS

### 2.1 Primary reagents

Ginsenoside Rg3 (purity 98.63%) (Dalian Fusheng Pharmaceutical Co., Ltd.), RPMI 1640 cell culture medium, trypsin–EDTA digestive solution, and transwell chambers were purchased from Corning, USA; fetal bovine serum was purchased from Gibco, USA; penicillin, streptomycin, RIPA protein lysate, BCA protein concentration assay kit, hypersensitive ECL kit, and horseradish peroxidase‐labeled goat antirabbit or goat antimouse IgG were purchased from Beyotime Biotechnology; phosphatase inhibitors were purchased from Calbiochem, Germany; propidium iodide (PI) was purchased from BD, USA; MTT and DMSO were purchased from Amrosco, USA; mouse antihuman GAPDH monoclonal antibodies were purchased from Elabscience, Wuhan, China; and mouse antihuman PTEN, p‐PI3K, AKT, and P53 antibodies were bought from Abcam.

### 2.2 Cells and culture

SGC‐7901 cells were provided by the ATCC. SGC‐7901 cells were cultured in RPMI 1640 medium containing 10% fetal bovine serum, 100 U/mL penicillin, and 100 μg/mL streptomycin in an incubator at 37°C and 5% CO_2_. The cells were passaged or used for subsequent experiments when the cell confluence reached 70%–80%.

### 2.3 MTT assay

SGC‐7901 cells were harvested and seeded in 96‐well culture plates at 5 × 10^5^ cells/well and cultured overnight in an incubator; the next day, ginsenoside Rg3 diluted in DMSO was added to the cells to final concentrations of 10, 20, and 40 mg/L with an equal volume of DMSO as a control, and duplicate wells set for each group. Twenty‐four hours later, 20 μl of MTT solution was added to each well, and the plates were incubated for an additional 4 hr; then, the supernatant was discarded, and 100 μl of DMSO was added to each well to dissolve the products, and the optical density of the solution at 490 nm was determined using a Tecan M200 fully automatic microplate reader (TECAN, Switzerland). The experiment was repeated three times.

### 2.4 Flow cytometry assay for cell apoptosis

Each group was prepared in a cell suspension at a concentration of 5 × 10^5^ cells/L, inoculated into a disposable culture flask, and cultured at 37°C in an atmosphere containing 5% CO_2_. The cells were also treated with ginsenoside Rg3 at final concentrations of 10, 20, and 40 mg/L with an equal volume of DMSO as the control, and 3 duplicate wells were set for each group. The cells in each group were treated for 24 hr, collected, centrifuged at 1,500 *g* for 5 min, then rinsed with PBS, fixed with 70% ethanol at 4°C for 12 hr, rinsed with PBS again, centrifuged at 1,000 *g* for 5 min, washed with PBS again, centrifuged at 1,000 *g* for 5 min, and resuspended with PBS in a tube dedicated for flow cytometry examination. Then, 0.5 ml of propidium iodide working solution was added to each tube and incubated at 37°C for 15 min in the dark, and the percentage of apoptotic cells was detected at a wavelength of 488 nm in triplicate.

### 2.5 Flow cytometry for cell cycle analysis

SGC‐7901 cells (5 × 10^5^ cells/well) in the logarithmic growth period were collected and inoculated into a 6‐well culture plate. When the cells reached 60%–70% confluence, ginsenoside Rg3 was added to each well at a final concentration of 10, 20, or 40 mg/L, with an equal volume of DMSO as a control, and 3 duplicate wells set for each group. Each group of cells was treated as specified for 24 hr; then, the cells were collected, washed twice in prechilled PBS, then fixed with 1 ml of 70% ethanol, centrifuged, and washed twice more with PBS. Then, 1 × 10^5^ cells were filtered through a 300‐mesh nylon membrane and collected in a 1.5 ml centrifuge tube, resuspended in 400 μl PI solution and incubated in the dark for 15 min, then loaded into a flow cytometer for cell cycle analysis.

### 2.6 Transwell assay

Transwell inserts were placed in a 24‐well plate. SGC7901 cells were harvested and inoculated into the upper chambers of the transwell inserts at 1 × 10^5^ cells/well. Culture medium containing 1% fetal bovine serum was added to the upper and lower chambers of the transwell cells. After 4 hr of culture, the culture medium in the lower chamber was discarded and replaced with culture medium containing 10% fetal bovine serum, and the upper chamber was replaced with culture medium containing 1% fetal bovine serum and ginsenoside Rg3 diluted with DMSO (final mass concentration 10, 20 and 40 g/L), and the same volume of DMSO in fresh culture medium was used as the negative control without ginsenoside Rg3 treatment. Each treatment was conducted in triplicate. After another 48 hr of culture, the upper chamber was removed, the extra liquid aspirated, and the cells that failed to migrate through the membrane wiped off with a wet cotton swab. Cells that migrated to the lower side of the membrane were fixed with 4% paraformaldehyde solution at room temperature for 20 min and stained with 0.1% hematoxylin for 8 min. Then, the membrane was washed with tap water, sealed with neutral gum, and observed and photographed under a light microscope, and the number of migrated cells was analyzed with ImageJ software. The experiment was repeated three times.

### 2.7 Wound‐healing assay

SGC‐7901 cells were seeded in 6‐well culture plates at 1 × 10^5^ cells/well, and when a monolayer formed, the medium was replaced with serum‐free medium to starve the cells for 4 hr. A straight line was drawn with a sterilized 200 μl pipette tip along the diameter of the wells, and the exfoliated cells were washed off with PBS. The cells were then treated with ginsenoside Rg3 at final concentrations of 10, 20, and 40 mg/L, with an equal volume of DMSO as a negative control. Three duplicate wells were set for each group. The width of the scratch was measured at the same place at the time of scratching (0 hr) and 24 hr after scratching using an inverted microscope (Olympus). ImageJ software was used to analyze the scratch width and calculate the mobility. The wound‐healing rate (%) was calculated as (scratch width at 0 hr − scratch width at 24 hr)/scratch width at 0 hr × 100%, which reflects lateral migration of the MGC‐803 cells. The experiment was repeated three times.

### 2.8 WB assay

SGC‐7901 cells were harvested and seeded in 6‐well plates at 5 × 10^5^ cells/well and cultured overnight. The next day, ginsenoside Rg3 was added to each well at a final concentration of 10, 20, or 40 mg/L, with an equal volume of DMSO as the negative control. After 48 hr of treatment, the SGC‐7901 cells of each group were harvested and lysed with RIPA lysis buffer containing phosphatase inhibitor to extract total protein. The protein concentration was determined using the BCA method, and 35 μg of total proteins was separated by 8% SDS‐PAGE. The protein bands were then transferred to a PVDF membrane, which was blocked with 5% skim milk at room temperature for 2 hr, incubated with mouse antihuman PTEN (1:500), p‐PI3K (1:500), AKT (1:500), P53 (1:500), or GAPDH (1:1,000) monoclonal antibody at 4°C overnight, washed and incubated with horseradish peroxidase‐labeled goat antirabbit or goat antimouse IgG (1:1,000) at room temperature for 2 hr, washed again, and visualized using ECL reagents and the ChemiDoc imaging system (Bio‐Rad). The gray values of the target protein bands were analyzed using Image Lab software. The relative expression level of each protein was represented as the ratio of the gray value of the target protein band to the gray value of the corresponding GAPDH band. The experiment was repeated three times.

### 2.9 Immunofluorescence assay to determine the effect of ginsenoside Rg3 on nuclear transport of p‐PI3K in SGC7901 cells

A suspension of SGC‐7901 cells was prepared, 5 × 10^5^ cells were inoculated in each well, and each group of cells was treated as specified for 48 hr. Then, the coverslip was removed, washed 3 times with PBS for 5 min, fixed with 4% paraformaldehyde at room temperature for 15 min, washed with PBS again 3 times for 5 min, aspirated to dryness, blocked with goat serum at 37°C for 1 hr, dried with filter paper, and incubated with primary antibody in a humidified chamber at 4°C overnight. The next day, the coverslips were rewarmed at 37°C for 1 hr, washed 3 times with PBS for 5 min, and incubated with FITC‐conjugated secondary antibody, incubated at 37°C for 1 hr, and washed with PBS 3 times for 5 min. For the nuclear stain, 0.4 μg/μl DAPI solution was used to cover the cells and incubated at room temperature for 2–5 min; then, the cells on the cover slip were washed 3 times with PBS for 5 min and sealed with glycerol for observation under a fluorescence microscope.

### 2.10 Statistical analysis

Analysis of the experimental data was carried out in SPSS 19.0. The relative data are shown as mean ± *SD* (standard deviation), comparisons of the means of multiple groups were performed by one‐way analysis of variance, and pairwise comparisons were performed using the LSD method. *p* < .05 was considered statistically significant.

## 3 RESULTS

### 3.1 Ginsenoside Rg3 affects cell proliferation

**FIGURE 1:**
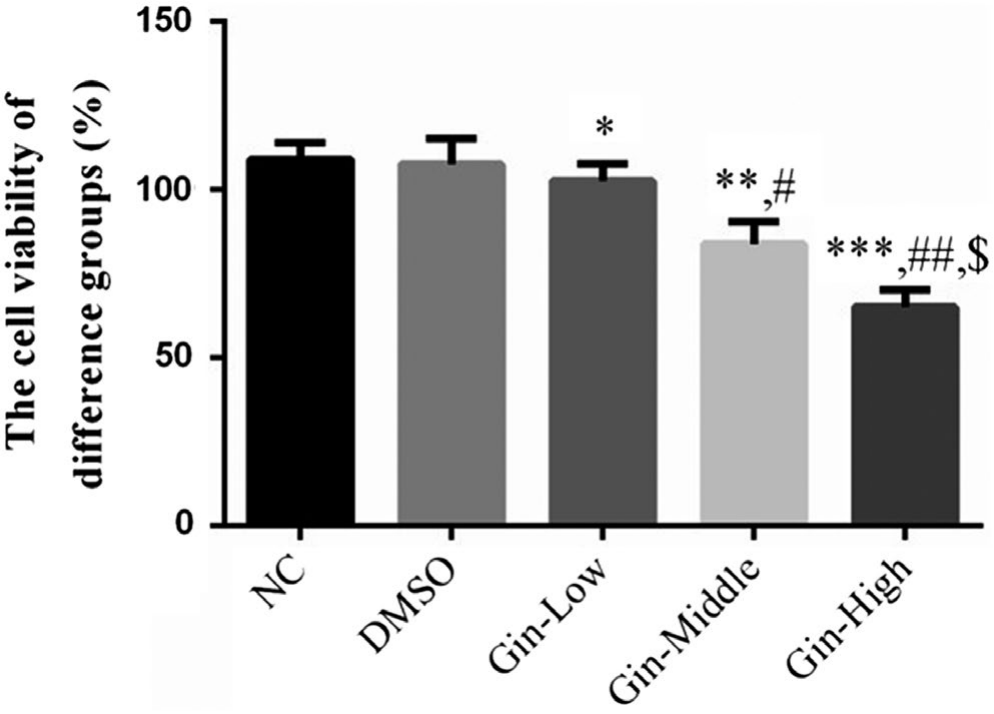
Ginsenoside Rg3 affects SGC‐7901 cell proliferation by MTT assay. *p < .05; **p < .01; ***p < .001, versus NC group; #*p* < .05; ##*p* < .01, versus Gin‐Low group; $*p* < .05, versus Gin‐Middle group

There were no significant differences in the cell proliferation rates of SGC‐7901 cells treated with DMSO only (*p* > .05, Figure 1); however, the cell proliferation rates of ginsenoside Rg3‐treated groups were significantly depressed (*p* < .05, Figure 1) in a dose‐dependent manner (*p* < .05, Figure 1).

### 3.2 Ginsenoside Rg3 affects SGC‐7901 cell apoptosis

**FIGURE 2:**
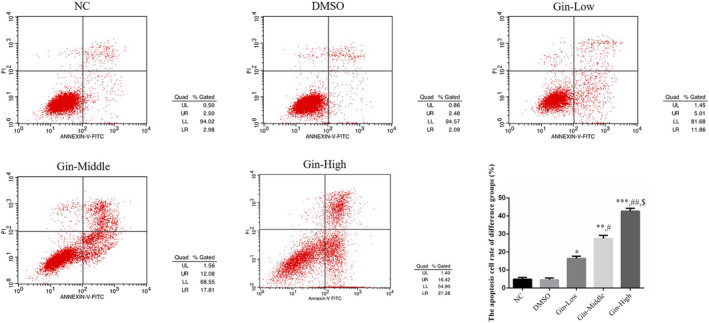
Ginsenoside Rg3 affects SGC‐7901 cell apoptosis by flow cytometry assay. *p < .05; **p < .01; ***p < .001, versus NC group; #*p* < .05; ##*p* < .01, versus Gin‐Low group; $*p* < .05, versus Gin‐Middle group

According to the results of flow cytometry, the apoptosis rates of the ginsenoside Rg3‐treated groups increased significantly (*p* < .05, respectively, Figure 2) in a dose‐dependent manner (*p* < .05, respectively, Figure 2), and the apoptosis rate in the DMSO group was not significantly different (*p* > .05, Figure 2). This result showed that DMSO had no effect on SGC‐7901.

### 3.3 Ginsenoside Rg3 affects the SGC‐7901 cell cycle

**FIGURE 3:**
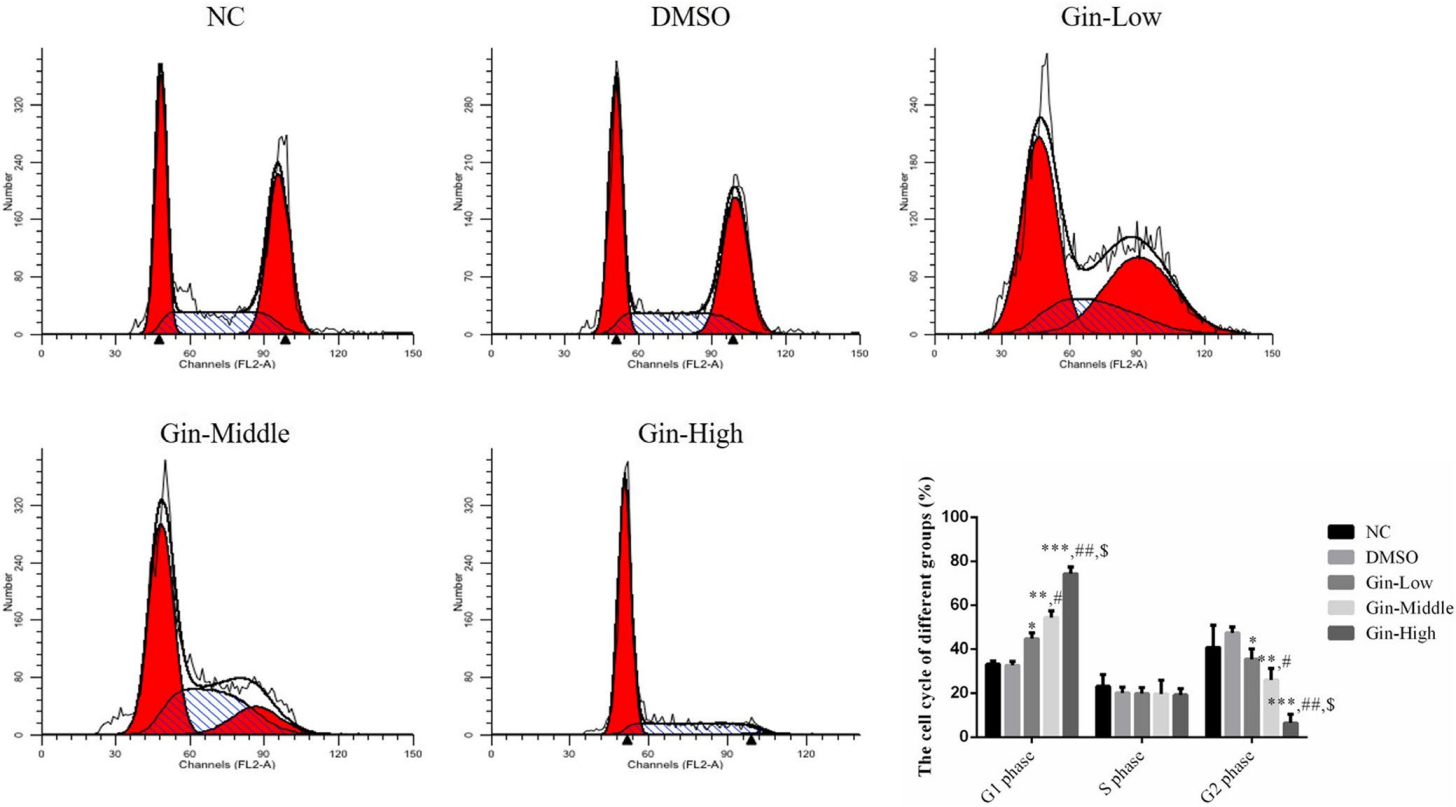
Ginsenoside Rg3 affects SGC‐7901 cell cycle by flow cytometry assay. *p < .05; **p < .01; ***p < .001, versus NC group; #*p* < .05; ##*p* < .01, versus Gin‐Low group; $*p* < .05, versus Gin‐Middle group

The proportion of cells in the G1 and G2 phases in the ginsenoside Rg3‐treated groups differed significantly (*p* < .05, Figure 3) in a dose‐dependent manner (*p* < .05, respectively, Figure 3). However, there were no significant differences between the NC and DMSO groups in the proportions of cells in the G1 and G2 phases.

### 3.4 Ginsenoside Rg3 affects SGC‐7901 cell invasion

**FIGURE 4:**
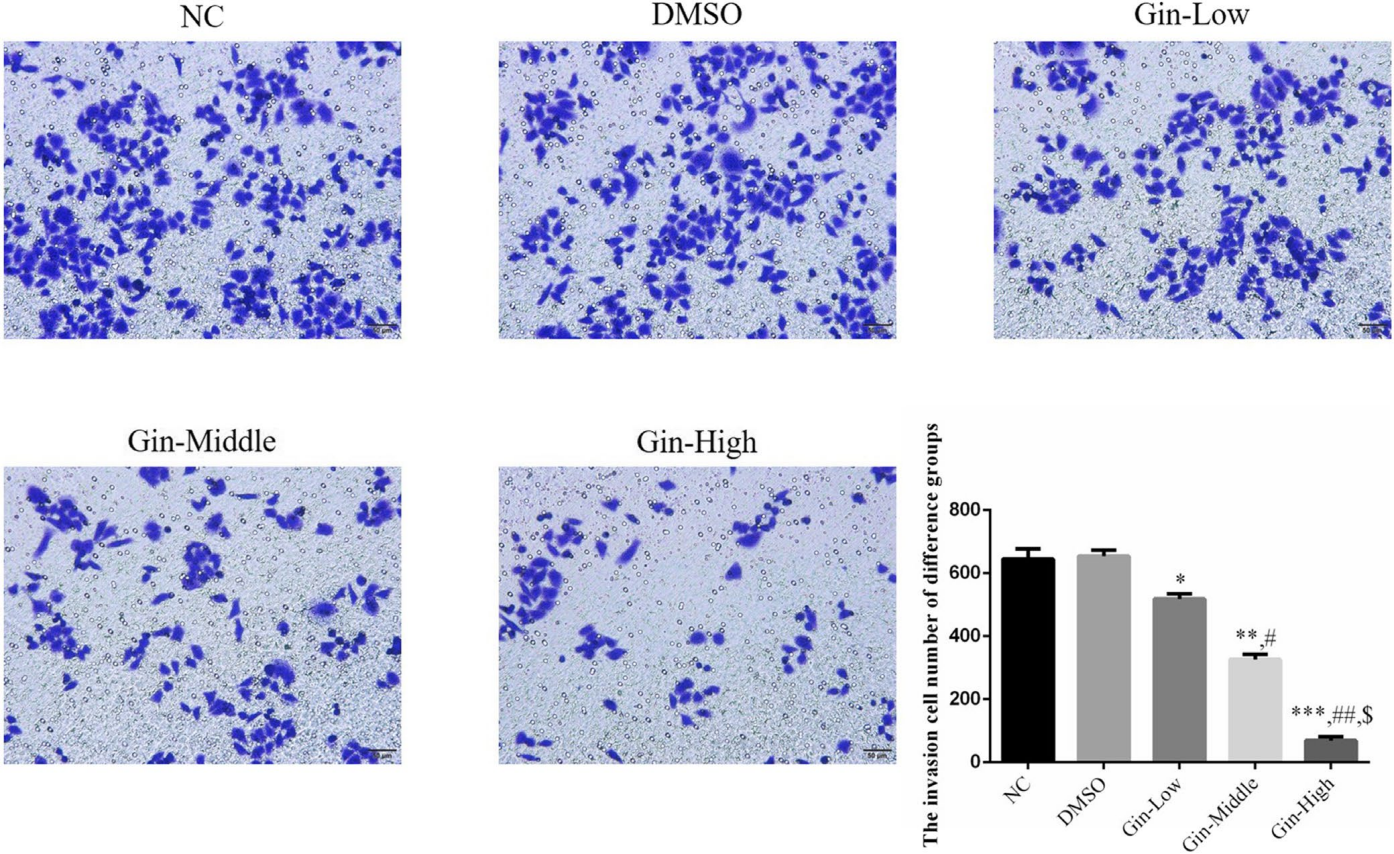
Ginsenoside Rg3 affects invasion SGC‐7901 cell number by transwell assay (×200). *p < .05; **p < .01; ***p < .001, versus NC group; #*p* < .05; ##*p* < .01, versus Gin‐Low group; $*p* < .05, versus Gin‐Middle group

The SGC‐7901 cell invasion numbers in the ginsenoside Rg3‐treated groups were significantly reduced (*p* < .05, respectively, Figure 4) compared with that of the NC group in a dose‐dependent manner (*p* < .05, Figure 4). Meanwhile, there were no significant differences between the NC and DMSO groups in the SGC‐7901 cell invasion number (*p* > .05, Figure 4).

### 3.5 Ginsenoside Rg3 affects SGC‐7901 cell migration

**FIGURE 5:**
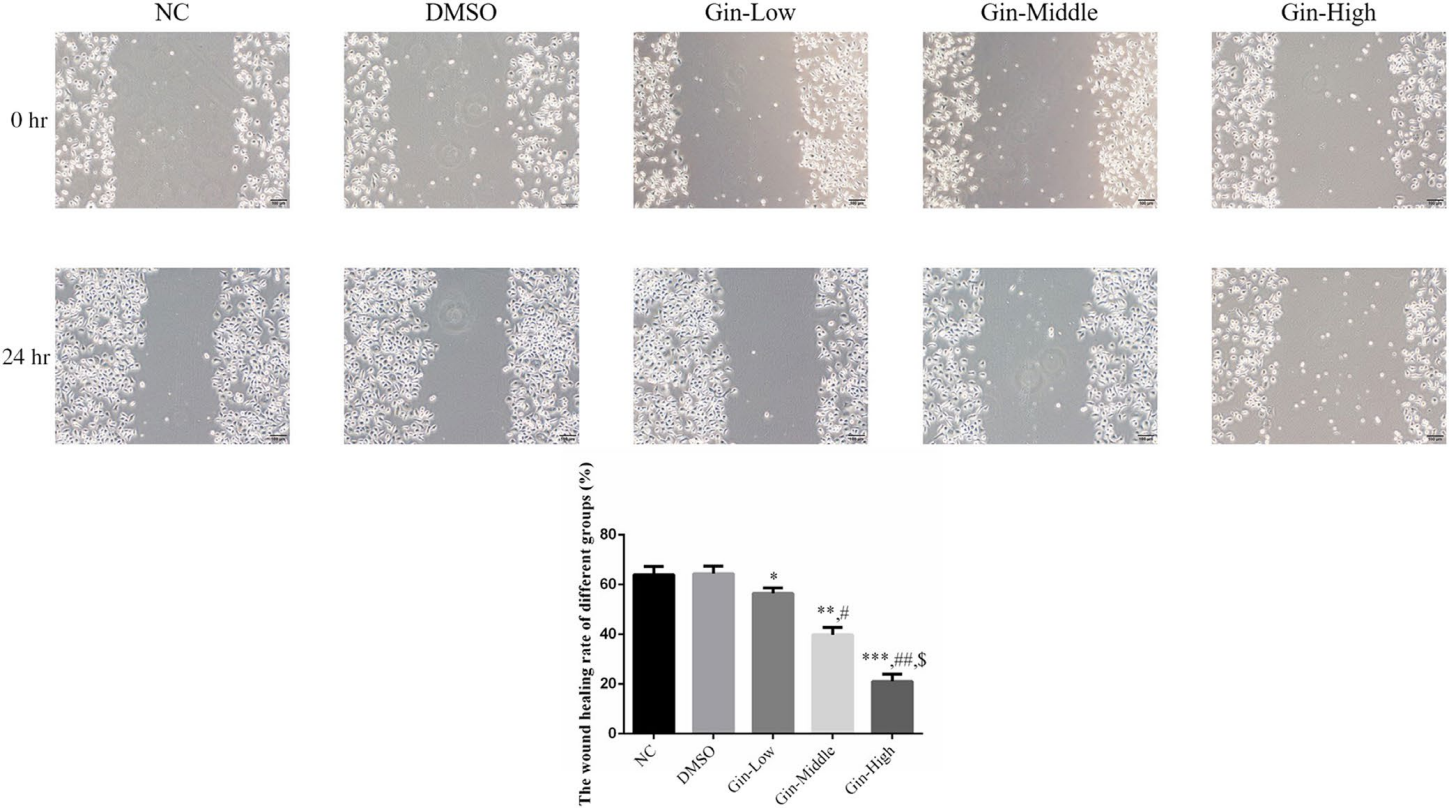
Ginsenoside Rg3 affects wound‐healing rate of difference groups by wound‐healing assay (×200). *p < .05; **p < .01; ***p < .001, versus NC group; #*p* < .05; ##*p* < .01, versus Gin‐Low group; $*p* < .05, versus Gin‐Middle group

The wound‐healing rates in the ginsenoside Rg3‐treated groups were significantly reduced (*p* < .05, respectively, Figure 5) compared with that of the NC group in a dose‐dependent manner (*p* < .05, Figure 5). Meanwhile, there were no significant differences in the wound‐healing rate between the NC and DMSO groups (*p* > .05, Figure 5).

### 3.6 Ginsenoside Rg3 affects relative protein expression

**FIGURE 6:**
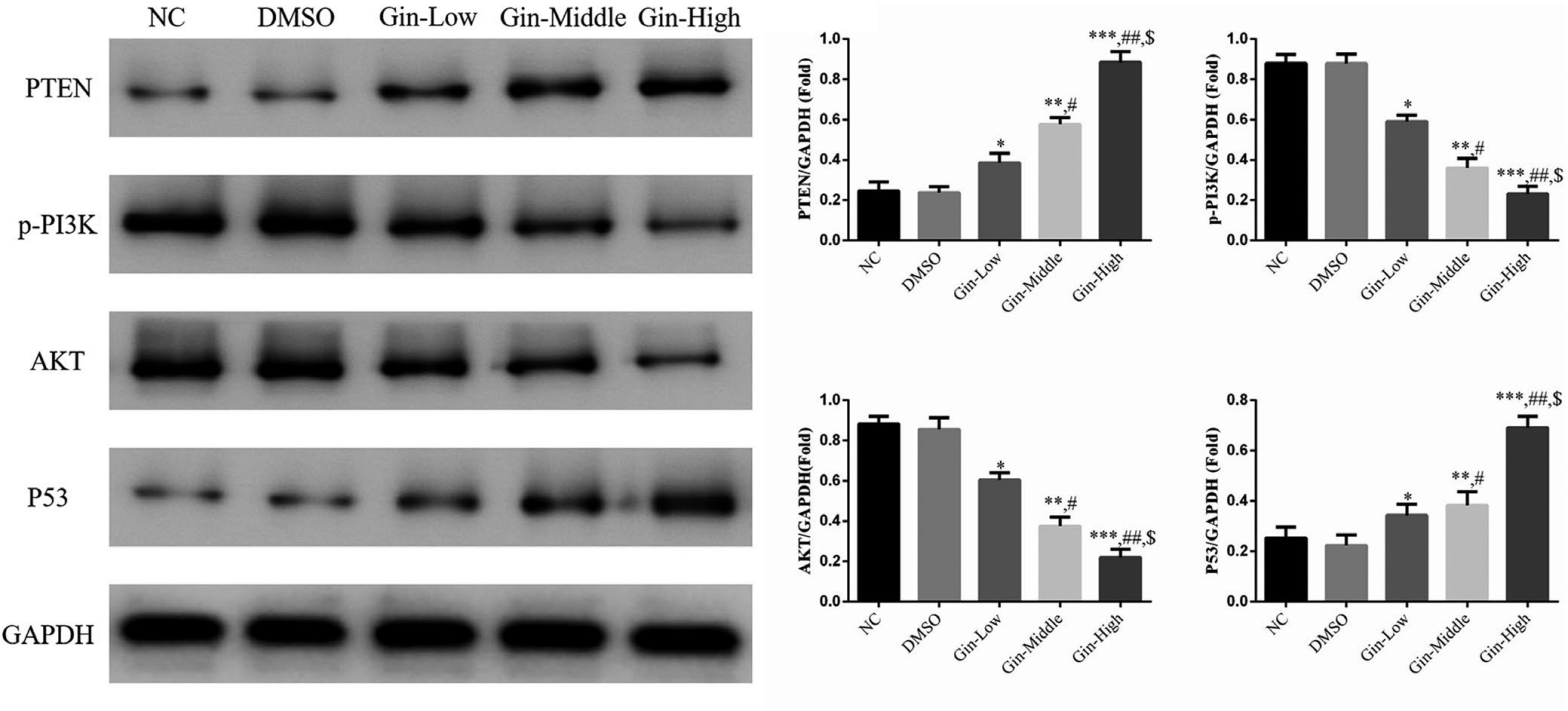
Ginsenoside Rg3 affects relative proteins expression by WB assay. *p < .05; **p < .01; ***p < .001, versus NC group; #*p* < .05; ##*p* < .01, versus Gin‐Low group; $*p* < .05, versus Gin‐Middle group

PTEN and P53 protein expression levels were significantly increased, and p‐PI3K and AKT protein expression levels were significantly depressed (*p* < .05 Figure 6) in the ginsenoside Rg3‐treated groups compared with the NC group in a dose‐dependent manner (*p* < .05, Figure 6). There were no significant differences between the NC and DMSO groups in PTEN, p‐PI3K, AKT, and P53 protein expression levels (*p* > .05, Figure 6).

### 3.7 Ginsenoside Rg3 affects p‐PI3K nuclear volume according to cellular immunofluorescence

**FIGURE 7:**
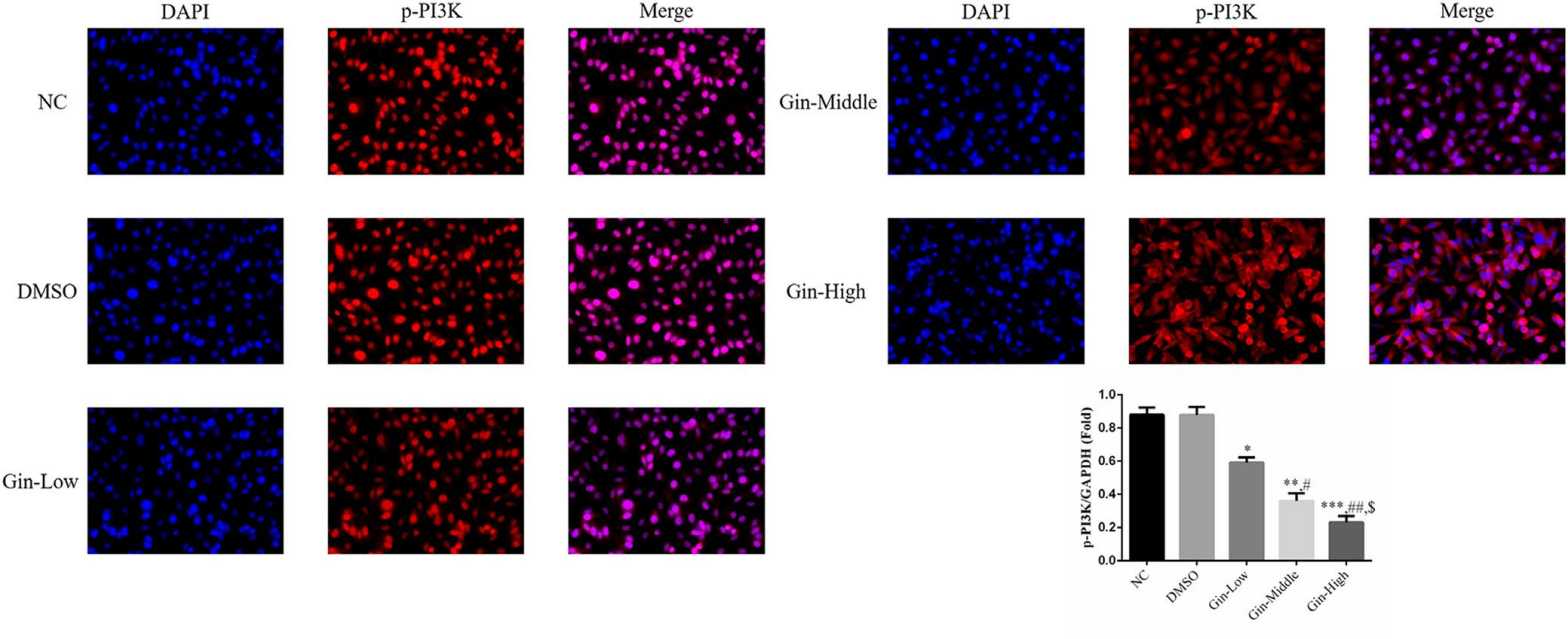
Ginsenoside Rg3 affects p‐PI3K nuclear volume by cellular immunofluorescence (×200). *p < .05; **p < .01; ***p < .001, versus NC group; #*p* < .05; ##*p* < .01, versus Gin‐Low group; $*p* < .05, versus Gin‐Middle group

According to cellular immunofluorescence, p‐PI3K nuclear volume was significantly depressed (*p* < .05, Figure 7) in the ginsenoside Rg3‐treated groups compared with the NC group in a dose‐dependent manner (*p* < .05, respectively, Figure 7). There were no significant differences between NC and DMSO in p‐PI3K nuclear volume (*p* > .05, Figure 7).

## 4 DISCUSSION

Ginseng contains saponins, polysaccharides, peptides, fatty acids, mineral oils, and other active ingredients (Chang et al., 2014; Jiang, Chen, Chen, & Zheng, 2011; Joo et al., 2015). Given these active ingredients, the pharmacology and immunostimulatory activity of ginsenoside Rg3 are obvious, such as antimutational, antistress, and antiaging effects, as well as the ability to improve the physiological and immune functions of the human body (Jiang et al., 2011). Our present study showed that ginsenoside Rg3 can effectively inhibit the biological activity of SGC‐7901. To explore the mechanism involved in the effects of ginsenoside Rg3 on SGC‐7901 cells, we further examined the expression of related proteins.

PTEN is a member of the dual specificity protein phosphatase (DSP) family; as such, it possesses both phospholipase and protein tyrosine phosphatase activities (Worby & Dixon, 2014). It acts as an antitumor gene by inhibiting the PI3K/AKT signaling pathway that promotes tumor growth (Sheng et al., 2019). Loss or mutation of the PTEN gene can lead to accelerated cell proliferation, decreased apoptosis and changes in cell migration and morphology, all of which contribute to tumor formation (Dillon & Miller, 2014). At present, abnormalities in PTEN have been confirmed to be closely related to the development, progression, and prognosis of glioma (Zhang et al., 2019), NSCLC (Shi et al., 2019), breast cancer (Xing et al., 2019), colon cancer (Liu et al., 2019), and other tumors. AKT is the central hub of the PI3K/AKT signaling pathway essential for cell survival, playing an important role in mediating the signals related to tumorigenesis and the survival mechanism of cells. Our results demonstrated that treatment with ginsenoside Rg3 significantly reduced the biological activity of SGC‐7901 cells by increasing PTEN expression and decreasing PI3K/AKT signaling activity. Meanwhile, as shown by immunofluorescence assay, the nuclear accumulation of PI3K was also inhibited. These may be the mechanisms involved in the inhibitory effect of ginsenoside Rg3 on the biological activity of SGC‐7901 cells.

Antitumor drugs kill tumor cells mainly by inducing apoptosis, which is often mediated by the P53 gene. PTEN induces apoptosis of tumor cells by inhibiting the PI3K/AKT signaling pathway, leading to an increased proportion of cells in the G1 phase, thereby enhancing P53 functioning (Delma et al., 2019; Han, 2019; Zhao et al., 2019). In our study, treatment of SGC‐7901 cells with ginsenoside Rg3 significantly increased p53 expression, leading to significant retention of SGC‐7901 cells in G1 phase and a high rate of apoptosis.

It can be concluded that ginsenoside Rg3 can effectively inhibit the biological activity of SGC‐7901 cells, and such an effect may be closely related to regulation of the PTEN/PI3K/AKT signaling pathway.

## CONFLICT OF INTEREST

None.
